# Where are the links? Using a causal loop diagram to assess interactions in healthcare coordination for youth experiencing homelessness in Toronto, Canada

**DOI:** 10.1186/s12961-024-01104-y

**Published:** 2024-01-30

**Authors:** Alzahra Hudani, Janet Long, Ronald Labonté, Sanni Yaya

**Affiliations:** 1 Interdisciplinary School of Health Sciences, University of Ottawa, Ottawa, Canada; 2 Centre for Healthcare Resilience and Implementation Science, Australian Institute of Health Innovation, Macquarie University, Sydney, Australia; 3 School of Epidemiology and Public Health, University of Ottawa, Ottawa, Canada; 4 School of International Development and Global Studies, University of Ottawa, 120 University Private, Ottawa, ON K1N 6N5 Canada

**Keywords:** Emergency youth shelter, Health system, Youth, Homelessness, Hospital, Healthcare coordination, Endless loop, Causal loop diagram, Variables, Links

## Abstract

**Background:**

Youth experiencing homelessness (YEH) suffer from poorer physical and mental health outcomes than stably housed youth. Additionally, YEH are forced to navigate fragmented health and social service systems on their own, where they often get lost between systems when transitioning or post-discharge. Inevitably, YEH require support with health system navigation and healthcare coordination. The aim of this study is to understand interactions within and between the emergency youth shelter (EYS) and health systems that affect healthcare coordination for YEH in Toronto, Canada, and how these interactions can be targeted to improve healthcare coordination for YEH.

**Methods:**

This study is part of a larger qualitative case study informed by the framework for transformative systems change. To understand interactions in healthcare coordination for YEH within and between the EYS and health systems, we developed a causal loop diagram (CLD) using in-depth interview data from 24 key informants at various levels of both systems. Open and focused codes developed during analysis using Charmaz’s constructivist grounded theory methodology were re-analysed to identify key variables, and links between them to create the CLD. The CLD was then validated by six stakeholders through a stakeholder forum.

**Results:**

The CLD illustrates six balancing and one reinforcing feedback loop in current healthcare coordination efforts within the EYS and health systems, respectively. Increasing EYS funding, building human resource capacity, strengthening inter and intra-systemic communication channels, and establishing strategic partnerships and formal referral pathways were identified among several other variables to be targeted to spiral positive change in healthcare coordination for YEH both within and between the EYS and health systems.

**Conclusions:**

The CLD provides a conceptual overview of the independent and integrated systems through which decision-makers can prioritize and guide interventions to strengthen healthcare coordination within and between the EYS and health systems. Overall, our research findings suggest that key variables such as streamlining communication and improving staff–youth relationships be prioritized, as each of these acts interdependently and influences YEH’s access, quality and coordination of healthcare.

## Introduction

Youth between the ages of 16–24 years comprise about 11% of the homeless population in Toronto, Canada, and are overrepresented by Indigenous, gender-diverse, racialized, refugee and newcomer youth [1]. According to “Without a Home”, the first national youth homelessness survey in Canada (*N* = 1103), 29.5% of youth participants identified as LGBTQ2S+^1^, 30.6% identified as Indigenous and 28.2% identified as members of racialized communities. About 40% of YEH who participated in the survey were younger than 16 years when they first experienced homelessness, and 10% were born outside of Canada [2].

Youth experiencing homelessness (YEH) are exposed to a myriad of factors that lead to poor health, including inadequate nutrition and sleep, poor hygiene, high levels of stress, increased sexual activity with more partners and increased exposure to infectious diseases [2–4]. Although YEH suffer from a variety of poor health outcomes, challenges with mental health and addictions are most prevalent [5, 6]. According to the most recent national youth homelessness survey (2019; *N* = 1375), 74% of youth respondents report high levels of distress, 35% report attempting suicide at least once and 33% report a drug overdose requiring hospitalization [7]. Additionally, YEH often suffer from physical and mental health comorbidities, and require support with health system access, navigation and coordination of health services [8–10]. Rarely do youth have the motivation, support and knowledge required to access the most appropriate health services for their needs [10]. Further, although many YEH migrate to Toronto to access specialized supports and services such as for emergency shelter, mental health and addictions, and education and employment supports, the systems providing these services are largely fragmented and operate in silos [11, 12].

Fragmentation of health and social services force YEH, many of whom have complex needs, to navigate these elaborate service systems on their own. This transient youth population rarely receives continuous care, and often get lost within and between these systems – this is also the case with the emergency youth shelter (EYS) and health systems in Toronto [13]. Although some shelter providers are attempting to integrate health services into EYSs with some success, there is still a disconnect in healthcare coordination for many YEH. Disintegrated system structures, insufficient funding and healthcare coordination processes and inhibiting policies such as information-sharing and privacy policies are among several factors that hinder healthcare coordination within and between these two systems. These factors intercept pathways to prevention response and timely healthcare, sometimes leading youth to inevitably experience health crisis such as overdosing, alcohol poisoning and suicide attempt. When experiencing crises, youth often enter a vicious cycle, which we refer to as the endless shelter–hospital loop, where youth are sent to receive emergency care at hospitals, are prematurely discharged into the EYS system and then re-enter the health system for similar health concerns/crises [14]. This critical disconnect requires strengthening healthcare coordination within and between systems, to improve the healthcare trajectories and health outcomes of YEH.

The Agency for Healthcare Research and Quality define healthcare coordination as “the deliberate organization of patient care activities between two or more participants, (including the patient), involved in a patient’s care to facilitate the appropriate delivery of health services” [15, 16]. In this context, we elaborate further by considering healthcare coordination at a systems and service level. At a systems level, healthcare coordination entails systems-level integration, which consists of centralized management and funding. At the service level, it can involve the coordinated delivery of health services within and/or across the EYS and health systems, and/or vertically or horizontally within system-based agencies [17]. Healthcare coordination for YEH, whether at a system and/or service level, has the potential to improve safety, accessibility and continuity of health services for YEH, and can reduce costs within both systems. Additionally, a fundamental element required in healthcare coordination for YEH is caring for patients holistically, and in doing so addressing the broader determinants of health [18].

The primary research question guiding this study is: “What interactions within and between the EYS and health systems affect healthcare coordination for YEH in Toronto?” By bridging systems science with grounded theory methodology, we aim to assess interactions between various elements involved in healthcare coordination within and between the EYS and health systems in Toronto, particularly through a causal loop diagram (CLD). These interactions consist of causal links within and between system elements, system feedback and self-regulation resulting from these links and interaction delays that may hinder efforts to strengthen healthcare coordination within and between these two systems. In developing a comprehensive illustration of these interactions, we aim to identify elements that can be targeted by policy-makers, shelter providers and other system-wide decision-makers within the two systems. To our knowledge, this is the first study to apply a systems-thinking and organizational change lens to the issue of fragmented healthcare coordination between the EYS and health systems for YEH.

## Methods

### Study design and setting

This research is part of a larger qualitative case study exploring engagement in healthcare coordination between the EYS and health systems in the inner-city and inner-suburban regions of Toronto. The case study is informed by the framework for transformative systems change developed by Foster-Fishman and colleagues, through which we first defined the boundaries of the Toronto-based EYS and health systems in their healthcare coordination roles, and then analysed fundamental system parts (that is, norms, resources, regulations and operations) that affect healthcare coordination within and between both systems [19]. This article is guided by the third and subsequent component of the framework, “assessing system interactions”. To understand interactions in healthcare coordination within and between the EYS and health systems, we developed a CLD– a systems map known to help understand long chains of consequences through system(s) behaviour, and through which we can speculate potential interventions [20]. Through the CLD, we explore causal links between key system variables, reinforcing and balancing interdependencies between these variables (that is, feedback loops), and interaction delays in healthcare coordination within and between the EYS and health systems [19].

### Data collection

#### Participants and recruitment

The CLD is informed by 24 semi-structured, in-depth key-informant interviews, which were facilitated virtually over Microsoft Teams between May 2021 and March 2022. Most interviews ranged between 45 min and 1 h in length. Interview participants were recruited using purposive and snowball sampling. First, purposive sampling was used to recruit high-level executives and clinicians who worked within either. We then used snowball sampling to recruit lower-level executives (for example, case managers) and frontline staff with clinical (for example, social workers and nurses) and non-clinical (for example, program coordinators and outreach counsellors) backgrounds. A few frontline staff working at EYSs and within the community health sector were asked to support with youth recruitment by sharing flyers and information sheets with youth who met inclusion criteria, and/or by posting flyers at their respective organizations. Youth were considered for an interview if they ranged between 16 and 24 years of age, resided at an EYS in Toronto and navigated healthcare integrated within an EYS or the broader health system. Youth who were interested in participating in the study emailed, texted or called the principal investigator (AH) to learn more and/or schedule a date and time for the interview. In total, AH facilitated interviews with eight key informants employed within the EYS system, seven within the health system, three whose work overlapped between systems and six youth.

#### Interview materials

Two separate interview guides were developed for youth and non-youth participants. Interview questions were open-ended and focused on identifying and describing healthcare coordination processes within and between the EYS and health systems in Toronto, from the diverse perspectives of interview participants.

### Data analysis

We analysed interview transcripts as part of the broader case study using three layers of coding, as informed by Charmaz’s constructivist grounded theory methodology [21]. Using NVivo 12.0 software, we used open coding to identify processes and actions relevant to healthcare coordination within and between the EYS and health systems. These codes were then organized into defined categories through focused coding. We examined each of these codes to determine key variables and causal links between them for the CLD. Links between variables infer causality and are represented by directional arrows between key variables. Positive (+) signs were assigned to arrow handles to denote a change in variables that occurred in the same direction (for example, an increase in variable A causes an increase in variable B), and negative signs were assigned to denote a change in the opposite direction (for example, an increase in variable A causes a decrease in variable B) [22, 23].

Theoretical coding was then used to integrate variables on the basis of causal links identified between them, and any feedback loops and delays that were determined thereafter. Two types of feedback loops emerged when variables connected to form closed loops, known as balancing, and reinforcing loops. Reinforcing feedback loops (R) are characterized as having spiralling effects, which can push a system out of balance, and balancing loops (B) stabilize the system [22, 23]. Interaction delays were denoted using double lines (||) that intercept causal links and demonstrate that a shift in one variable will likely have a delayed impact on another variable – these often make systems change efforts look unsuccessful [19]. The CLD was created using Vensim PLE software.

### Trustworthiness

An iteration of the CLD was developed by AH, and then critically discussed with six stakeholders through a 1-h allotment of a 2-h virtual stakeholder engagement forum. Four stakeholders were interviewed participants, and two were recommended by stakeholders and met inclusion criteria. Two YEH had confirmed attendance to the forum but were unable to make it. AH presented the CLD as a story during the first half of the forum, after which participants were asked to reflect and share input on the diagram by responding to the following questions:

1. Are there any missing variables? Do any of the variables need to be renamed or replaced?
2. Any there any places where connections are absent or weak, and/or are needed?
3. Are there any challenges that could interfere with strengthening links in the CLD?

Verification of the CLD through stakeholder engagement adds rigour to the findings presented by helping mitigate bias and increasing confidence in the final iteration of the diagram. Several links were verified, and new links were discussed and created.

### Ethical approval

Ethical approval for the study was granted by the University of Ottawa Health Sciences and Sciences Research Ethics Board (Ethics file number: H-12-20-5771).

## Results

**Fig. 1:**
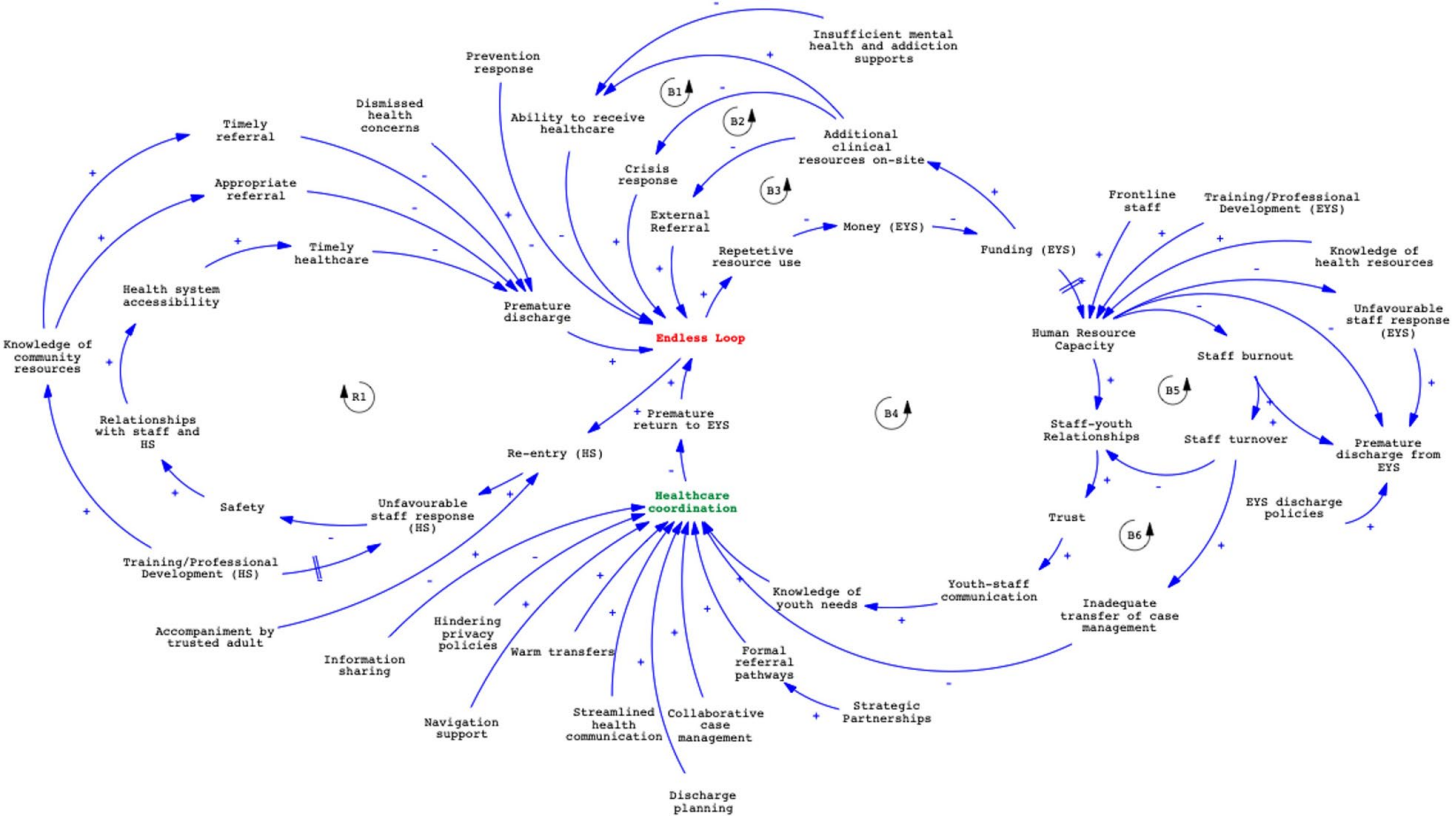
Causal loop diagram displaying links in healthcare coordination within and between the EYS and health systems

The CLD is divided to illustrate key links in healthcare coordination within the EYS system (right) and within the health system (left) and elements that may strengthen coordination within and between both systems (bottom centre). Healthcare coordination and the endless loop are central to the diagram, as the former is the goal and purpose of this research, while the latter is analysed to be one of the most significant, adverse and spiralling outcomes resulting from barriers in healthcare coordination. Several variables comprising the causal links are environmental factors that can potentially strengthen healthcare coordination for YEH once targeted. Overall, funding, human resource capacity, strength of staff–youth relationships, collaborative case management and communication are dominant variables within and/or across these two systems, which have the potential to support YEH with their healthcare coordination needs once targeted and bridge the EYS and health systems in their efforts coordinating care for this population. Findings for this article are presented as a narrative review of the CLD illustrated in Fig. 1.

### The funding problem

Underfunding within the EYS system interferes with the requisite human resource capacity-building needed within and between systems. Frontline staff often experience vicarious trauma, are overburdened and do not always have sufficient training, skills or knowledge to serve this population in ways that benefit their trajectories to healthcare. Challenges with underfunding directly spiral to challenges and barriers in coordinating healthcare, and inevitably the endless shelter–hospital loop as depicted through feedback loops B1–B6. These feedback loops demonstrate the need for increased funding within EYSs to integrate additional clinical resources on-site. Integration of clinical resources such as primary care, psychiatric counselling and harm reduction supports may cause: (1) healthcare to be more accessible for YEH (B1), (2) mitigation of crisis response to hospital emergency departments where YEH often have negative experiences and get lost within the systems (B2) and (3) lessened need for EYS staff to refer YEH externally to receive healthcare, where they often undergo challenges with follow-up and supporting continuity of healthcare due to inhibiting privacy and information-sharing policies, and incomplete or missing health documentation/discharge notes (B3).

In addition to underfunding, money within and between these systems are inequitably distributed. Within EYSs, some YEH have access to more extensive healthcare resources and coordination support depending on where they are residing. An executive at Shelter C shares her concern:

*We’re a service provider for the City of Toronto and shelter services, and they have a set of shelter standards that lay out all the things that we are required to provide to young people. The city provides us with a certain amount of funding - that funding, never meets [the standards] and we are always forced to fundraise to top that up… but then you look at organizations like [Shelter A] and they have an incredibly robust fundraising program, and they can offer a huge variety of [health] services without support from the city because of their fundraising.*

She elaborates on the inequity:

*There are a lot of things that are layered in [the funding problem]. Part of it is that, if you work in a charity, there’s a perception of you work in a charity, you shouldn’t need all those things that people have when they work in banks, right. And I think that the things that people have when they work in banks are what make the banks work.*

Moreover, an executive at Shelter A shares a clear discrepancy in how EYSs are funded in comparison to community health centres which are also mandated to serve at-risk, vulnerable populations within the community: “Shelters should be funded like community health centres, [and] not forced to fundraise.”

### Relationship development and capacity-building

Relationship development is a recurring theme in strengthening healthcare coordination, whether intra-organizationally between staff and youth, intra-systemically between stakeholders within the EYS or health systems and inter-systemically between the EYS and health systems.

#### Significance of staff–youth relationships in healthcare coordination success

Entry into the EYS system is often an access point to healthcare coordination for YEH. Therefore, developing positive, strong, safe and trusting staff–youth relationships is prerequisite to motivating YEH to seek health services, and coordinating healthcare with them. A 23-year-old youth shares:

*When you’re in crisis, you need the support. And sometimes it is very hard to have that, whether it’s like, whatever staff is on duty can’t relate to you or can’t connect with you, or you just don’t feel comfortable with [them]. Or sometimes they just do not care. I’m just tired of it, and I think that’s the main concern.*

Establishing rapport and building trust with YEH early on during their shelter stay may empower them to share their health concerns with their case worker, including those that may be sensitive or require immediate attention – before they turn into crisis. A barrier to staff–youth relationship development consistently reported by interviewees, however, is the scarcity of human resource capacity experienced by most, if not all, EYSs in the region. EYSs are often short-staffed due to funding constraints causing some staff to wear multiple hats, exacerbating burnout. EYSs also tend to face frequent staff turnover, which interferes with staff–youth relationship development (B5) and poor transfer of case management to new staff (B6). Moreover, frontline staff sometimes do not have the training, skills and/or knowledge to identify the type of care that youth need from them nor do they have the tools required to engage with YEH and/or provide this care. These variables inevitably hinder staffs’ ability to coordinate healthcare with and for youth within the EYS system. Emphasis on inputs such as staff training and professional development may help staff build the skills needed to structure safety for YEH, collaboratively problem-solve with them, etc., and in doing so strengthen relationships between them, and better understand their health needs. Enhancing staffs’ knowledge and awareness of appropriate clinical and youth-based health resources in the community may also facilitate successful healthcare coordination for YEH, in addition to the other inputs shown in the CLD.

#### Positive staff–youth relations and health system accessibility for YEH

Premature discharge from hospitals and re-entry into the health system occur due to: (l) healthcare providers’ missing and/or dismissing mental health concerns, after which YEH re-experience crises and return to hospital emergency departments; (2) youth having negative experiences with health system staff, which prevents them from willingly and fully communicating their health concerns to staff, and/or trusting them with sensitive health information; (3) healthcare providers exhibiting their own biases towards the population; (4) healthcare providers perceiving YEH’s needs as being outside the scope of their care; and (5) healthcare providers not referring YEH to specialized or long-term healthcare when needed due to insufficient information to effectively diagnose youth and/or their lack of awareness about appropriate community-based health and social support resources to which they can refer or transfer youth. A social worker at an inner-city hospital shares:

*I work in the emergency department and one of the things that always comes up for me is the whenever there’s a new program or service introduced in the community, it’s usually communicated to leadership [or] management on some level and it takes a very long time, if at all, to get filtered down to the people who would actually be trying to make these referrals or connections...and I imagine most hospitals experience the same thing.*

Training and professional development for health system staff, and improvement in system-wide and organizational processes, are therefore significant inputs required to improve safety and accessibility for YEH once they enter or re-enter the health system (R1), and to help appropriately refer them to specialist and/or long-term care in a timely manner. Some training recommendations from interview participants include adopting organizational and system wide norms that facilitate dignified and trauma-informed care in healthcare settings, understanding intersectionality and applying this lens to youth patients’ contexts, and being familiar with current research (for example, the Street Needs Assessment) to keep updated with relevant statistics, and understand the layers and complexities of people’s experiences of homelessness that affect their health.

Moreover, accompaniment by a trusted adult to emotionally support and advocate for YEH when entering hospitals was suggested by a few key informants to help YEH receive the healthcare they need and prevent re-entry into the system. The accompanying adult should have sufficient background knowledge of youths’ health history and concerns to help fill health information gaps that need to be communicated to healthcare providers. A stakeholder forum participant explains:

*If there is a solid case manager or somebody that they know well from the shelter system, this would be the time to get that collateral information and support the patient particularly while they’re being triaged, because that’s the note that’s going to be read by the doctor. That’s a note that’s going to determine which area of the hospital they go to, whether it’s acute or ambulatory...if you can walk youth to the emergency and stay just, for the first half hour [because it] is very critical to get that information over to the triage nurse and get your contact information on file, so that you can be reached as the doctor sees them and as the consult starts to happen...the person at the housing where they’re staying is the one that’s seen them all day, every day. They know their baseline. They can speak to some of the symptoms in a way that the youth may not be able to articulate, as sort of a support person.*

This type of support was expressed as a critical input to strengthen youths’ access and coordination of healthcare, in an otherwise poorly accessible environment where youth may cycle through many different triggers and emotions during their visit. However, this input is only applicable if YEH have a trusted adult in their life who they consent to accompany them – and the example provided is inevitably dependent on staff–youth relationships developed within the EYS system. Further, a frontline staff working at an inner-city hospital explains the benefit of having an outreach department within the hospital. She shares:

*I would love to see every hospital have an outreach department. I think it allows for those multiple visits, it allows for an extension of relationships, and it allows you a chance to really get to know somebody, to be able to work with them effectively, to be able to address multiple social issues that are contributing to their [youths’] physical health. So, it kind of sees somebody through the entirety of who they are, rather than just what they’re going through or where they’re living, right... I’d love to see hospitals never discharge anyone to the street.*

This approach to healthcare provision, where relationship-building and follow-up support are emphasized, may help healthcare teams understand the complexities and comorbidities that patients experiencing homelessness face, and in having this context, coordinate healthcare in a more intentional and informed way.

A few stakeholder forum participants also commented on how rapport and relationship building with YEH may be more successful outside institutional walls. Participants suggested that frontline staff facilitate warm transfers by accompanying youth to suitable partners and referral sites to transfer healthcare accountability to new healthcare providers and support with relationship-building between health system staff and youth. This was discussed as a strategy to potentially prevent youth from getting prematurely discharged or lost in the larger system post-transfer or post-referral.

### The need for formalized and strategic partnerships

Establishing formalized and strategic partnerships within and between EYS and health system institutions, agencies and organizations may help system staff more easily and appropriately refer YEH to external healthcare. Currently, most referral pathways depend on informal connections between sectors and system staff’s pre-existing connections. These partnerships are inconsistent across organizations, and do not always comprise of community health services that are best suited for the diverse needs of YEH (for example, harm reduction and support for LGBTQ2S + youth). Some strategic partnerships have been developed through Ontario Health Teams and are believed to be a step in the right direction. However, more formal partnerships are needed, whether for health services to be integrated on-site at EYSs or for frontline staff to appropriately refer their youth clients.

### Improving processes and adjusting policies to enhance communication

Enhancing communication intra- and inter-organizationally and systemically may help fill significant gaps in coordinating healthcare and supporting youths’ continuity of care. Some examples of how to do this as suggested by stakeholders within both systems include: collaborative case management; integrating a single, streamlined and used-friendly platform to allow staff to share detailed health information and discharge plans; having designated staff within organizations/institutions for follow-up and information-sharing; and coordinating warm collaborations and transfers for YEH.

Although these suggestions are promising in theory, several barriers exist in enhancing inter-systemic communication and resource-sharing. First, information-sharing and privacy policies must be adjusted to allow health information custodians within youths’ circle of care to share and access critical health information – if they receive consent from youth. Information sharing between service providers is pivotal, as YEH are often subject to complex situations and should have detailed discharge plans in place. Key informants employed at EYSs and a community health centre explain that YEH do not always return from the hospital with discharge papers, and if they do, they are often not detailed enough to support youths’ continuity of care. In other cases, YEH do return with discharge documents, but the plans in place are not always accessible options for youth due to their respective circumstances – for example, healthcare providers prescribing medication to youth who do not have coverage. Enhancing communication channels through adequate information-sharing has the potential to reduce health information gaps that are commonly encountered by healthcare providers, upon YEHs entry or re-entry into the EYS and/or health systems. As YEH are a transient and vulnerable population, having easily accessible and complete health documentation could help intake staff support youth with healthcare access and coordination early on, and reduce entrapment in the vicious shelter–hospital loop.

A second barrier to enhancing communication is the tension and resentment that appears to exist between system providers, which is seemingly triggered by structural systemic differences, and policies that hamper progression in youths’ trajectories to healthcare access and coordination. This was evident in key informant interviews and responses from stakeholder forum participants. A case manager at Shelter B shares:

*I think sometimes our inability to see our own flaws and weaknesses and sort of like placing the blame, let’s say, on a school or on a health provider or whatever – it gets in the way of progress, because there is a bit of tension sometimes between the different providers and some resentment. And I think without a genuine and authentic attempt to mend these issues, that could get in the way of any progress.*

Overall, improving intra- and inter-systemic communication and collaboration through policy and process amendments has the potential to strengthen system wide relationships and healthcare coordination for YEH.

## Discussion

It is evident that the variables/inputs illustrated in the CLD are interdependent and should therefore be considered holistically when thinking about or designing policy or program-based interventions. In the CLD, all six feedback loops shown on the EYS system side (right) are balancing, and the one feedback loop on the health system side (left) is reinforcing. We presume this may be due to the incredibly complex and high-barrier health system that has structurally evolved to have limited accessibility for vulnerable and marginalized populations, including YEH [24–26]. For instance, health concerns faced by people experiencing homelessness require consideration of the bio-psycho-social factors affecting their health, as opposed to simple clinical diagnoses [27, 28]. Evidence from this study and others show that hospital staff often fail to provide adequate mental health and addictions treatment for people experiencing homelessness, and that approaches to providing healthcare are not usually person-centred or trauma-informed [29, 30]. Further, evidence also indicates the need for equity-oriented healthcare services, whereby YEH who are newcomers and/or identify as members of LGBTQ2S+ , racialized and/or Indigenous communities have their individual health and social support needs met [31], ideally through a coordinated systems approach. These examples demonstrate two of several layers that would need to be targeted to improve accessibility, and thereby strengthen healthcare access and healthcare coordination for YEH. The EYS system is contrarily less complex and specialized to serve YEH who need emergency shelter. The feedback loops on the EYS system are goal-seeking and pinpoint key variables that can be targeted to improve healthcare coordination within the system. Although the EYS and health systems are mandated to provide services needed by YEH, neither are explicitly mandated to coordinate health services for this population – although healthcare coordination is critical in preventing re-entry into each system, and additional healthcare costs.

The issue of accountability in healthcare coordination for YEH was found to be a significant challenge requiring more attention, as was inferred in key informant interviews and the stakeholder forum. While healthcare coordination is proclaimed to be a critical task and process required within and between systems, there are not any policies that explicitly require staff within either of these systems to take responsibility in coordinating healthcare for this population. For instance, guidelines within the Toronto Shelter Standards are broad, with some regulation on case management, supports and services, where staff are expected to work with YEH to determine their immediate needs and concerns no later than 36 h after admission, including supports for mental health, substance use and harm reduction (p. 65–66) [32]. In section 10.2 of the Toronto Shelter Standards, there are some regulations around health and mental health, where staff are required to support clients with finding appropriate support services and referring YEH to these supports as needed [32]. The challenge with these expectations, however, is that the EYS system does not have the funding or capacity to deliver on these according to the diverse needs of their client population, especially those who are higher risk and have more complex needs. As the primary funders of EYSs in Toronto, our study results suggest that the Shelter, Support and Housing Administration consider the key inputs required to help shelter operators act on these guidelines (for example, equitable funding and increasing human resource capacity through staff training and professional development, privacy and information-sharing policy amendments). One healthcare provider within the health system expressed that health concerns experienced by YEH are socially rooted and so healthcare coordination may go beyond the current scope and mandate of their roles. The obscurity around who is accountable in coordinating healthcare for YEH has led to resentment and finger-pointing between system stakeholders and has inevitably affected relationship-building, which is a key variable in improving healthcare coordination within and between systems.

Most stakeholders within EYSs and the community health sector have experienced frustrations with coordinating healthcare and agree that more emphasis and direction need to be placed on strengthening healthcare coordination, integrating healthcare within the EYS system, incorporating outreach roles at inner-city hospitals, and integrating systems to improve healthcare accessibility. An EYS executive director commented on how EYSs often must beg healthcare organizations to come in to serve YEH. To strengthen healthcare coordination through systems integration, she suggests pairing EYSs with community health centres, as they are also mandated to serve populations on the basis of community need.

Overall, employing a systems-thinking lens to the issue of poorly coordinated healthcare within and between the EYS and health systems has helped strengthen our understanding of the interconnectedness of this wicked social issue, and the need for shared responsibility across these public systems and others to tackle them. For instance, youth who are experiencing homelessness and mental health issues are likely to interact with multiple systems such as housing, healthcare, education and justice – and accordingly the responsibility of their care should be spread across many government systems respectively overseeing them. Both mental health and housing status are inherently linked and connected to broader structural conditions such as poverty. The intersection of structural issues and policy fields indicates the need for interventions to take a systems approach to produce tangible results in the lives of YEH. By spearheading interventions using a systems approach and ensuring that interventions including policies and services are well coordinated, there is potential for positive outcomes in other areas through targeted investment in any part of the EYS system, health system and/or both systems [33].

### Strengths and limitations

While many scholarly sources in Canada and internationally examine barriers and facilitators to healthcare access for YEH and recommend strengthening healthcare coordination [34–36], this is the first to apply a systems-thinking lens to the multi-faceted issue. The framework for transformative systems change encouraged us to preliminarily examine each system’s layers, niches, organizations and actors and, in doing so, learn about health programs and services that cater to the needs of YEH and systems integration efforts to improve healthcare access for YEH. Bounding the systems and analysing each system to understand the deeper structural elements involved in coordinating healthcare for YEH helped build a strong foundation for the CLD, building selectivity and transparency into the study design [37]. Additionally, the CLD considers the collective perspectives of stakeholders across various levels of each system, enhancing its accuracy and validity.

Although the perspectives of YEH are reflected in the CLD, receiving their input and feedback at the stakeholder forum would have increased confidence in the final iteration of the diagram. Additionally, we found that the guiding questions asked to refine and validate the CLD at the forum could have been simplified to engage stakeholders further. For instance, the CLD was described as a story, and similarly could have been examined to elucidate gaps and challenges in the story, rather than using technical jargon to ask about variables and links between them. Finally, it is important to note that CLDs are static representations of interactions between elements, which may change over time. While we have extrapolated key variables from key informant interview data, it is unfeasible to capture all elements involved in the two complex adaptive systems [38].

## Future directions

As part of the final component of the framework for transformative systems change, “identifying levers for change”, [19] we explored more targeted and tangible solutions to enhance healthcare coordination within and between the EYS and health systems for YEH. These interventions were discussed in depth as part of the second half of the stakeholder forum and are presented in a final article culminating this case study.

## Conclusion

The CLD provides a conceptual overview of the independent and integrated systems through which decision-makers can prioritize and guide interventions to strengthen healthcare coordination for YEH. Overall, and on the basis of our reported findings, we recommend that priority be given to variables/inputs such as funding, building human resource capacity and enhancing intra- and inter-systemic communication. Each of these variables should be considered holistically, as they are interdependent and influence access, quality and coordination of healthcare for YEH. Additionally, strengthening healthcare coordination for YEH may help improve their health outcomes, which are interconnected with various other social determinants of health.

## Data Availability

Anonymized interview audio recordings and/or transcripts are available from the corresponding author on reasonable request.

## Abbreviations

YEH: Youth experiencing homelessness
EYS: Emergency youth shelter
CLD: Causal loop diagram

## Footnotes

1 Refers to people who identify as lesbian, gay, bisexual, transgender, queer or questioning, two-spirited and other sexual identities.
